# Supplementary material for: Neuromorphic photonic networks using silicon photonic weight banks
Source: Sci Rep. 2017 Aug 7;7:7430. doi: 10.1038/s41598-017-07754-z (PMC5547135; doi:10.1038/s41598-017-07754-z)
Supplement: Supplementary file 1 — Supplementary info [file 41598_2017_7754_MOESM1_ESM.pdf]

Neuromorphic photonic networks using silicon photonic weight banks

Supplementary information

Alexander N. Tait, Thomas Ferreira de Lima, Ellen Zhou, Allie X. Wu, Mitchell A. Nahmias,
Bhavin J. Shastri, and Paul R. Prucnal

1 Derivation of Theoretical Bifurcations

The CTRNN model of interest is described by equations (1-2). Mathematical analysis of dynamical systems begins by examining fixed states (where $\dot{\vec{s}} = 0$) and the effects of parameters on their behavior. Bifurcations occur when the number or stability of fixed-points change as a result of parameter variation. Here, we derive simple bifurcations of small networks[38]. Predictions of amplitude and frequency are then compared to experiment in Fig. 2 and Fig. 3. For the sake of analysis, the neuron transfer function, whether sigmoidal (in the case of a perceptron) or sinusoidal (in the case of a MZM neuron), is approximated to the first nonlinear term: $\sigma(s) \approx \alpha s - \kappa s^3$, where α and κ are positive coefficients.

Cusp bifurcations describe transitions from monostability to bistability. A cusp can be observed in the simplest case of equations (1-2), which is a single node with self-feedback weight, W_F.

$$0 = W_F \sigma(s^*) - \frac{s^*}{\tau} + w_{in} u^* = \kappa W_F s^{*3} - \left(\alpha W_F - \tau^{-1}\right) s^* - w_{in} u^* \tag{S1.1}$$

where s^* and u^* are the steady-state scalar values of the neuron state and input, respectively.

When the input is zero, the steady-state solutions have the form:

$$s^*_{(1)} = 0; \quad s^*_{(2,3)} = \pm\sqrt{\frac{\alpha}{\kappa}\frac{W_F - W_B}{W_F}} \tag{S1.2}$$

where $W_B = (\alpha\tau)^{-1}$ is the bifurcation weight, and subscripts index the three solution branches. Below W_B, solution branches (2) and (3) are imaginary and therefore do not physically exist. This expression, plotted on the W_F-y axis of Fig. 2(b), exhibits the standard form of a pitchfork bifurcation, in which two stable solution branches arise out of one stable branch.

Returning to the general expression, the inputs, u^*, that yield steady-state solutions, s^*, take the form

$$u^* = \frac{\kappa W_F}{w_{in}} \left(s^{*3} - \frac{\alpha}{\kappa}\frac{W_F - W_B}{W_F} s^* \right) \tag{S1.3}$$

The resulting, familiar S-shaped bistable curve is plotted on the u-y axis of Fig. 2(b). Three roots of s^* exist when feedback weight is fixed above the pitchfork bifurcation value. The edges of this bistable regime are referred to as saddle-node points because the unstable middle saddle and one of the stable nodes annihilate one another. The saddle-node points (s^*_{SN}, u^*_{SN}) are found where the derivative of u^* in equation (S1.3) is zero with respect to s^*.

$$s^*_{SN} = \pm\sqrt{\frac{\alpha}{3\kappa}\frac{W_F - W_B}{W_F}} \ ; \quad u^*_{SN} = \pm\frac{2}{3}\frac{\alpha^{3/2}}{w_{in}\sqrt{3\kappa W_F}} \left(W_F - W_B\right)^{3/2} \tag{S1.4}$$

These parametric equations define a cusp, which is projected onto the W_F-u axis of Fig. 2(b). The cusp bifurcation is more informative than either the pitchfork or saddle-node bifurcations because it is described only in reference to two parameters, while the other bifurcations can occur in systems of one parameter.

Hopf bifurcations are characterized by a transition from stable to oscillating dynamics. In experiments and this analysis, we fix the off-diagonal weights asymmetrically such that $-w_{12} = w_{21} = 1$ and parameterize the diagonals such that $w_{11} = w_{22} = W_F$. Under this formulation, there is always one and only one steady-state at $\vec{s} = 0$. To examine its stability, we linearize the system around this point to yield the Jacobian matrix whose eigenvalues indicate fixed-point stability.

$$\mathbf{J} = \frac{d}{ds}\left(\frac{ds}{dt}\right) = \alpha \begin{bmatrix} W_F - (\alpha\tau)^{-1} & -1 \\ 1 & W_F - (\alpha\tau)^{-1} \end{bmatrix}; \quad \text{eigenvalues: } \lambda = W_F - (\alpha\tau)^{-1} \pm i \tag{S1.5}$$

The imaginary part of the eigenvalue pair is indicative of oscillating behavior. The real part of the eigenvalue switches sign at the bifurcation weight $W_B = (\alpha\tau)^{-1}$. In this case, when the only fixed-point solution becomes unstable, a stable limit cycle arises instead of new stable states. Near threshold, we can assume a circular form of the limit cycle in order to model its expected amplitude, A, and frequency, ω.

$$s_1(t) = A\sin(\omega t); \quad s_2(t) = A\cos(\omega t) \tag{S1.6}$$

At points where ωt is a multiple of 2π, the time-derivative of s_2 is zero. Examining the $\dot{s}_2$ equation from equations (1-2),

$$\left.\frac{ds_2}{dt}\right|_{\omega t = 2\pi m} = 0 = \sigma(0) + W_F\sigma(A) - \tau^{-1}A \tag{S1.7}$$

$$A = \sqrt{\frac{\alpha}{\kappa}\frac{W_F - W_B}{W_F}} \tag{S1.8}$$

where m is an integer. The amplitude follows a form similar to that of the pitchfork bifurcation in equation (S1.2) and is plotted as a red curve on the s_1-W_F axis of Fig. 3(d). The equation for $\dot{s}_1$ at this same point can be used to find the angular frequency.

$$\left.\frac{ds_1}{dt}\right|_{\omega t=2\pi m} = -A\omega = W_F\sigma(0) - \sigma(A) \tag{S1.9}$$

$$\omega = \frac{\tau^{-1}}{W_F} \tag{S1.10}$$

The expected limit cycle frequency is therefore finite at the Hopf point and inversely proportional above, as shown in Fig. 3(e).

2 NEF Compilation Procedure

2.1 Solving ODEs with Photonic Modulator Neurons

The complete jupyter notebook used to generate plots in this section and in Fig. 4 is available in: https://github.com/lightwave-lab/Neuromorphic_Silicon_Photonics

Modifications to the nengo project

Nengo is based exclusively on monotonic, non-negative output neuron models. However, its encoding-decoding algorithms should work with other kinds of neuron models. Here, we use the following `FourierSinusoid` class of neurons included in our fork of the nengo project.

The Lorenz chaotic attractor

In this simulation, we chose to construct a neural network using the neurons defined above to solve a classical chaotic dynamical system named "Lorenz attractor".

The equations are:

$$\dot{x}_0 = \nu(x_1 - x_0) \qquad \dot{x}_1 = x_0(\rho - x_2) - x_1 \qquad \dot{x}_2 = x_0 x_1 - \beta x_2$$

Since x_2 is centered around approximately ρ, and since NEF ensembles are usually optimized to represent values within a certain radius of the origin, we substitute $x_2' = x_2 - \rho$, giving these equations:

$$\dot{x}_0 = \nu(x_1 - x_0) \qquad \dot{x}_1 = -x_0 x_2' - x_1 \qquad \dot{x}_2' = x_0 x_1 - \beta(x_2' + \rho)$$

Refer to the standard example of the Lorenz attractor solver with 2000 neurons in a nengo example. *Note that the last equation for x_2' is typically shown with an error in that example and in other articles from Prof. Eliasmith's group.

2.2 Encoding strategy

From here onwards, we will refer the Lorenz system in its reduced form as as $\vec{x} = f(\vec{x})$, with:

$$\vec{x} = [x_0, x_1, x_2']^T \quad \text{and} \quad f(\vec{x}) = \begin{bmatrix} \nu(x_1 - x_0) \\ -x_0 x_2' - x_1 \\ x_0 x_1 - \beta(x_2' + \rho) \end{bmatrix}$$

In the following sections, we briefly explain the details on how nengo can be used to inform us on how to configure a photonic neural network to emulate an accelerated ODE, having the Lorenz attractor as an example.

Neuron model

Using nengo, we instantiate a population of N neurons that are all-to-all interconnected. These neurons are responsible of *representing* the vector $\vec{x}$ at any time t. We consider the state of each neuron as $\vec{s} = [s_i]$ for neuron i. The ODE that models each neuron, in this case, is:

$$\tau s_i + s_i = u_i$$

where u_i represents the post-synaptic input of the neuron and $y_i = \sigma(s_i)$ its output.

Nengo encoding strategy

In order to *encode* a vector $\vec{x}$ in the population N, nengo performs the following linear transformation (it has to be linear for the method to work):

$$s_i = g_i \vec{e}_i \cdot \vec{x} + b_i$$

where g_i is a gain term, $\vec{e}_i$ is an encoder vector, and b_i is a bias term. This is called the *encoding strategy*.

Nonlinear operations are effectively performed by linear combinations of the neural nonlinearities $\sigma(s_i)$. Therefore, it is the encoder's mission to generate as much entropy about the variables $\vec{x}$ as possible. This can be done by generating a diverse set of $(g, \vec{e}, b)$ parameters. Below, we do this by using $\vec{e}_i = [1, \pm 1, \pm 1]$, mixing all components of $\vec{x}$ together. Note: this can be optimized even further by noticing that the ODE does not contain $x_0 x_2$ terms.

Because we know that σ is a sinusoid, we create a set of (g, b) values to span a Fourier-like basis of functions across the domain $\vec{e}_i \cdot \vec{x} \in [-1, 1]$. (See tuning curves).

```python
# Intercept, in this case, corresponds to where the tuning curve intercepts
# zero. Range of [-.5, .5] corresponds to [-pi, pi]
ints = [0, 1/4]
# This number represents how many periods do we want between -1 and 1
# (see tuning curves below)
rats = s_pi * np.arange(1, 4)/2
# Encoder multipliers
enst = [-1,1]

num_intercepts = len(ints)
num_max_rates = len(rats)
num_encoders = len(enst) ** 2

j = 0
encoders = np.zeros(shape=(num_neurons, 3))
intercepts = np.zeros(num_neurons)
max_rates = np.zeros_like(intercepts)
for ir in range(num_max_rates):
    for ii in range(num_intercepts):
        for ie0 in range(len(enst)):
            if ie0 is 0:
                continue
            for ie1 in range(len(enst)):
                for ie2 in range(len(enst)):
                    vertex = np.array([enst[ie0], enst[ie1], enst[ie2]])
                    if not np.all(vertex == 0):
                        encoders[j,:] = vertex
                        intercepts[j] = ints[ii]
                        max_rates[j] = rats[ir]
                        j += 1
```

Neuron	Encoder	Intercept	Max_rates
1	[1. -1. -1.]	0.0	0.05
2	[1. -1. 1.]	0.0	0.05
3	[1. 1. -1.]	0.0	0.05
4	[1. 1. 1.]	0.0	0.05
5	[1. -1. -1.]	0.25	0.05
6	[1. -1. 1.]	0.25	0.05
7	[1. 1. -1.]	0.25	0.05
8	[1. 1. 1.]	0.25	0.05
9	[1. -1. -1.]	0.0	0.1
10	[1. -1. 1.]	0.0	0.1
11	[1. 1. -1.]	0.0	0.1
12	[1. 1. 1.]	0.0	0.1
13	[1. -1. -1.]	0.25	0.1
14	[1. -1. 1.]	0.25	0.1
15	[1. 1. -1.]	0.25	0.1
16	[1. 1. 1.]	0.25	0.1
17	[1. -1. -1.]	0.0	0.15
18	[1. -1. 1.]	0.0	0.15
19	[1. 1. -1.]	0.0	0.15
20	[1. 1. 1.]	0.0	0.15
21	[1. -1. -1.]	0.25	0.15
22	[1. -1. 1.]	0.25	0.15
23	[1. 1. -1.]	0.25	0.15
24	[1. 1. 1.]	0.25	0.15

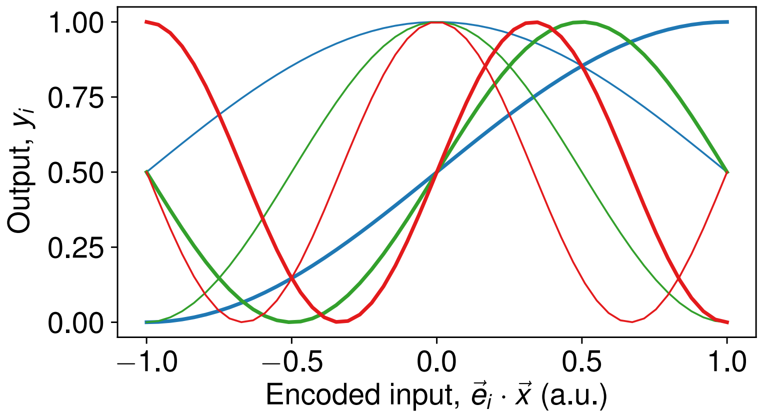

Figure S2.1. Selected tuning curves corresponding to sinusoidal Mach-Zehnder transfer functions. Output is transmission and input is voltage normalized to V_π. The encoded input is derived from the dot product of the encoding weight vector and the input signal vector.

Tuning curves in Fourier basis

Here, assuming that the neuron states are $s_i = g_i \vec{e}_i \cdot \vec{x} + b_i$, we plot the functions $\sigma(s_i)$ for neurons with different g_i, b_i values according to the previous table.

Timing

In order to account for the true-time delay between the output of a neuron and the feedback latency of the photonic waveguides, we instantiate a delay node in nengo, which essentially offsetstime signals in time.

```
# Round-trip feedback delay in ns
delayTime = .048
# gamma is a characteristic time scale in real time units
# The coefficient gamma/delayTime determines the stability
# In paper, coefficient was 65 (spurious), 104 (inaccurate), 260 (looks good)
gamma = 260 * delayTime

# We'll make a simple object to implement the delayed feedback
class Delay(object):
    def __init__(self, dimensions, timesteps=50):
        timesteps = max(timesteps,1)
        self.history = np.zeros((timesteps, dimensions))
    def step(self, t, x):
        self.history = np.roll(self.history, -1, axis=0)
        self.history[-1] = x
        return self.history[0]
delay = Delay(3, timesteps=int(delayTime / dt))
```

2.3 Nengo Implementation

After having the encoding strategy laid out, we are then ready to extract any function from the population. Here, we set the feedback function to be $\tau f(\vec{x}) + \vec{x}$, for reasons that are explained in the following section.

```
# the ODE to emulate
# The default values for sigma, beta and rho originally used by Lorenz.
# Cf. https://en.wikipedia.org/wiki/Lorenz_system#Analysis
nu = 10
beta = 8.0/3
```

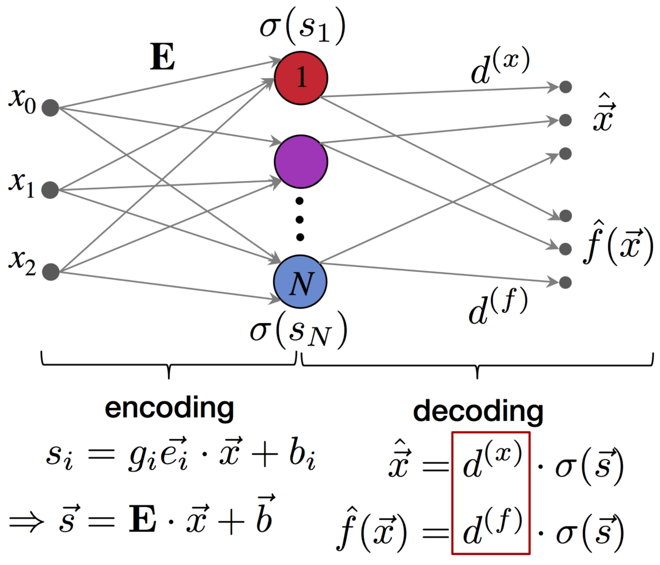

Figure S2.2. Encoding and decoding a variable with a population of neurons in the Neural Engineering Framework.

```
rho = 28
def feedback(x):
    dx0 = (-nu * x[0] + nu * x[1]) / gamma
    dx1 = (-x[0] * x[2] - x[1]) / gamma
    dx2 = (x[0] * x[1] - beta * (x[2] + rho)) / gamma

    return [dx0 * tau + x[0],
            dx1 * tau + x[1],
            dx2 * tau + x[2]]
```

In the following code snippet, we show how we can instantiate an ensemble of neurons and set the feedback connections to emulate the Lorenz attractor.

```
# The main ensemble
state = nengo.Ensemble(num_neurons, dimensions=3,
    intercepts=intercepts,
    neuron_type=nengo.neurons.FourierSinusoid(max_overall_rate=max_transmission,
                                               s_pi=s_pi),
    max_rates=max_rates,
    encoders=encoders, radius=60.)

# This special node calls a function every timestep,
# in this case a class method of delay
delay_node = nengo.Node(delay.step, size_in=3, size_out=3)

# Connections from state to delay and back
cdel = nengo.Connection(state, delay_node,
                        function=feedback, synapse=tau)
conn = nengo.Connection(delay_node, state)
```

2.4 Decoding strategy: calculating weight matrix

As mentioned, nengo decodes a function $h(\vec{x})$ from the population of neurons by a linear decoding strategy, i.e. a matrix $d^{(h)}$ resulting in an estimator $\hat{h}(\vec{x})$:

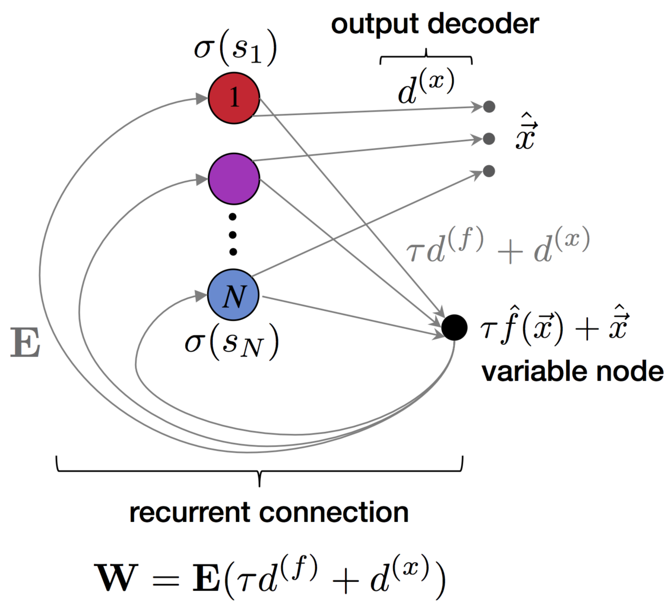

Figure S2.3. Recurrent connection for dynamical system emulation in the Neural Engineering Framework.

$$\hat{h}(\vec{x}) = d^{(h)}\vec{y}$$

where $y_i = \sigma(s_i) = \sigma(g_i\vec{e}_i \cdot \vec{x} + b_i)$.

This matrix $d^{(h)}$ is uniquely dependent on the encoder strategy, the neuron's transfer function σ and the function h. As a result, it can be pre-computed before any real-time simulation. Namely, it attempts to minimize the following objective function:

$$J = \int \left\| d^{(h)}\vec{y} - h(\vec{x}) \right\| \mathrm{d}\vec{x}$$

where the integral is over the desired range of values of $\vec{x}$.

The minimum can be calculated via the Moore-Penrose pseudoinverse method (Stewart et al. Front Neuroinform. 3 (2009)):

$$\Gamma_{ij} = \int y_i y_j \mathrm{d}\vec{x}$$

$$\Upsilon_i = \int y_i h(\vec{x}) \mathrm{d}\vec{x}$$

$$d^{(h)} = \Gamma^{-1} \cdot \Upsilon$$

Weight matrix

If we add an all-to-all recurrent connection to the neural population, their collective dynamics is described by the following ODE system:

$$\tau\dot{\vec{s}} + \vec{s} = \overline{\overline{W}}\sigma(\vec{s}) + \vec{I}$$

where $\overline{\overline{W}}$ is the weight matrix and $\vec{I}$ a bias vector.

Nengo sets $\overline{\overline{W}} = \overline{\overline{E}}(d^{(x)} + \tau d^{(f)})$ and $\vec{I} = \vec{b}$, where $\overline{\overline{E}}_{ij} = (\vec{e}_i)_j$. When applied to the ODE above, it is easy to see that one can recover the Lorenz system:

$$\overline{\overline{E}}(\tau\dot{\vec{x}} + \vec{x}) = \overline{\overline{E}}(\hat{\vec{x}} + \tau\hat{f}(\vec{x}))$$

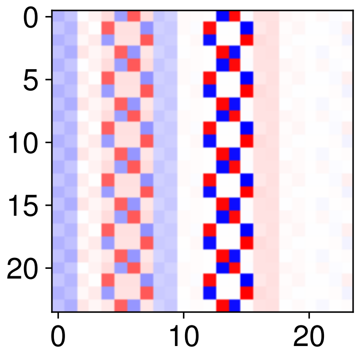

Figure S2.4. Recurrent weight matrix returned by Nengo compiler procedure. Red (blue) correspond to weights that are more positive (negative). X and Y axes correspond to neuron index, from 1 to 24.

$$\implies \dot{\vec{x}} = f(\vec{x}) + \varepsilon(\vec{x})$$

where $\varepsilon(\vec{x}) = (1/\tau)(\hat{\vec{x}} - \vec{x}) + \hat{f}(\vec{x}) - f(\vec{x})$.

Below, we show the computed weight matrix $\overline{\overline{W}}$ for this system.
